# Overexpression and translocation of dynamin 2 promotes tumor aggressiveness in breast carcinomas

**DOI:** 10.17179/excli2020-2762

**Published:** 2020-10-29

**Authors:** Roya Sajed, Leili Saeednejad Zanjani, Mandana Rahimi, Maryam Mansoori, Amir-Hassan Zarnani, Zahra Madjd, Roya Ghods

**Affiliations:** 1 Department of Molecular Medicine, Faculty of Advanced Technologies in Medicine, Iran University of Medicine Sciences (IUMS), Tehran, Iran; 2 Oncopathology Research Center, Iran University of Medical Sciences (IUMS), Tehran, Iran; 3 Hasheminejad Kidney Center, Pathology Department, Iran University of Medical Sciences (IUMS), Tehran, Iran; 4 Department of Immunology, School of Public Health, Tehran University of Medical Sciences (TUMS), Tehran, Iran; 5 Reproductive Immunology Research Center, Avicenna Research Institute (ACECR), Tehran, Iran

**Keywords:** Dynamin 2, breast cancer, immunohistochemistry, tissue microarray (TMA), invasion, cancer progression

## Abstract

Dynamin 2 is a GTPase protein that has been implicated in cancer progression through its various roles such as endocytosis, morphogenesis, epithelial-mesenchymal transition (EMT), cellular contractions, and focal adhesion maturation. The increased expression levels of this molecule have been demonstrated with the development of several cancers such as prostate, pancreas, and bladder. However, its clinical significance in breast cancer is unclear yet. In the present study, the membranous, cytoplasmic, and nuclear expression levels of dynamin 2 molecule were evaluated for the first time, using immunohistochemistry (IHC) on tissue microarray (TMA) slides in 113 invasive breast cancer tissues. Moreover, afterward, the association between the dynamin 2 expression and clinicopathological features was determined. Our finding showed that, a higher nuclear expression of dynamin 2 is significantly associated with an increase in tumor stage (*P = 0.05)*, histological grade (*P = 0.001*), and age of the patients (*P = 0.03*). In addition, analysis of the cytoplasmic expression levels of this molecule revealed that, there was a statistically significant difference between the expression levels of dynamin 2 among the different breast cancer subtypes (*P = 0.003*). Moreover, a significant association was found between the increased expression of dynamin 2 membranous and vascular invasion (VI) (*P = 0.02*). We showed that dynamin 2 protein expression has an association with more aggressive tumor behavior and more advanced disease in the patients with breast cancer; therefore, dynamin 2 molecule could be considered as an indicator of disease progression and aggressiveness.

## Introduction

Breast cancer is the most common cancer among women worldwide. In the United States, this cancer accounts for 30 % of all cases and 15 % of all the cancer-related deaths among women (Siegel et al., 2019[39]). Despite the advances in the treatment of breast cancer such as surgery, radiotherapy, and chemotherapy; drug resistance and metastasis are known as major causes of death (Mansoori et al., 2019[27]). Thus, this kind of cancer is a serious public health problem resulting in high mortality and morbidity rates. It has been shown that in cancer cells, finding some new molecules associated with the histopathological characteristics of cancer such as histological grade, tumor stages, and invasion helps determining the appropriate treatment for the disease and also prevents excessive costs (Blumen et al., 2016[2]).

Dynamin is a GTPase molecule, which was firstly introduced as a microtubule-binding molecule. Accordingly, it has a molecular weight of 96 kDa with three different isoforms (dynamin 1, 2, and 3), which are the proteins derived from the transcription of three genes, DNM1, DNM2, and DNM3, respectively. Dynamin has a tissue-specific expression, including isoform 2 that is continuously expressed in normal tissues while isoform 1 is significantly expressed in the neurons as well as isoform 3 that is expressed in the testis and postsynaptic nervous system (Ferguson and De Camilli, 2012[7]; Singh et al., 2017[40]). Moreover, Dynamin is localized on cell membranes (endoplasmic reticulum, Golgi apparatus, mitochondria, and cell membrane) and is also known as one of the essential components of vesicles involved in receptor-mediated endocytosis, caveolae internalization, vascular traffic inside, and outside the Golgi apparatus. However, the best known role for dynamin 2 is its essential role in the formation of clathrin-coated vesicles during endocytosis (Praefcke and McMahon, 2004[32]; Yoon et al., 1998[46]; Hinshaw, 2000[13]; Oh et al., 1998[29]). Therefore, dynamin using cadherin molecule endocytosis, which is a molecule that interconnects epithelial cells, can decrease the level of this molecule in cancer cells and also plays an important role in cancer progression by helping in the separation of cells from adjacent cells in the tissue (Paterson et al., 2003[30]). In addition, dynamin 2 also plays a role in morphogenesis, epithelial-mesenchymal transition (EMT), actomyosin contractions, and focal adhesion maturation (Chua et al., 2009[4]; Lamouille et al., 2014[23]; Edwards et al., 2016[5]; Jeong et al., 2006[15]; Gu et al., 2017[11]). 

In this regard, various studies have shown the increased levels of dynamin 2 through the development of various cancers such as pancreatic and prostate cancers (Eppinga et al., 2012[6]; Xu et al., 2014[42]). In addition, several studies have proved the importance of the dynamin 2 overexpression in increasing the invasion of lung cancer cells (Yamada et al., 2016[44]). Moreover, it has been shown that, Dynamin-Cortactin-Arp2/3 complex mediates actin reorganization, and the structure of the cytoskeletal actin also changes during the progression of cancer cells. Accordingly, these changes affect the phenotypes, survival, proliferation, invasion, metastasis, and activation of specific signaling pathways in the cell (Matsubara and Bissell, 2016[28]; Krueger et al., 2003[22]). In addition, investigations have shown that, dynamin inhibitors prevent the invasion of cancer cells by inducing caspase-mediated apoptosis (Yamada et al., 2009[44]; Joshi et al., 2011[16]). Also, a recent study indicated that, dynamin 2 inhibitor could be considered as a new therapeutic target for cervical cancer (Lee et al., 2016[25]).

Considering the above-mentioned findings, the importance of dynamin 2 molecule and its important role in the development of cancer have been identified; however, up to now, very little research has been done to link this molecule with breast cancer progression. 

In this study, we have evaluated the localization of expression of dynamin 2 in membranous, cytoplasmic, and nuclear sites of tumor cells for the first time in a series of the breast cancer tissue samples using immunohistochemistry on tissue microarray (TMA) slides, which were related to disease stage and histological grade as well as its aggressiveness.

## Materials and Methods

### Patient's characteristics and tumor samples

A total of 145 paraffin-embedded tissue blocks from the patients with breast cancer were collected from the Imam Khomeini Hospital in Urmia, Iran, from 2011 to 2016. Notably, none of these patients had received any treatment (chemotherapy or radiotherapy) prior to surgery. These samples comprised various subtypes of breast cancer including invasive ductal carcinoma (IDC), invasive lobular carcinoma (ILC), IDC and ILC, metaplastic as well as the other types of breast cancer. Moreover, the hematoxylin and eosin (H&E) stained slides and medical archival records were retrieved to access the clinicopathological parameters including age, tumor types, tumor side, tumor size (maximum tumor diameter), histological grade, tumor stage, lymph node involvement (LNI), and vascular invasion (VI). In this study, histological grading was defined in terms of the Bloom Richardson system (Bloom and Richardson, 1957[1]). Tumor stage was performed based on the pTNM classification for breast cancer. Furthermore, 10 whole section of normal tissues of the breast cancer samples were used related to women who had gone under surgery for mammoplasty, to compare the expression pattern and the distribution of dynamin 2 in a range of tissue specimens.

### Tissue microarray (TMA) construction

The breast cancer TMAs were prepared as it was described earlier (Kalantari et al., 2017[18]). Briefly, H&E slides were examined by a pathologist to select the most representative areas in different regions of the tumor. Afterward, the selected regions of blocks were punched out (0.6 mm diameter) and then transferred into a new recipient block using Tissue Arrayer Minicore (ALPHELYS, Plaisir, France). In the present study, three cores were evaluated from each tumor, which were then scored individually, thus the issue of heterogeneous antigen expression was overcome (Hoos et al., 2001[14]; Jourdan et al., 2003[17]; Langer et al., 2006[24]). Afterward, the obtained slides were prepared from TMAs blocks.

### Immunohistochemistry (IHC) staining for dynamin 2 protein expression

Briefly, TMA slides were deparaffinized, rehydrated, washed, and then endogenous peroxidase activity was blocked by 3 % H_2_O_2_ for 20 min at room temperature. Subsequently, the tissue slides were washed three times in Tris Buffered Saline (TBS). Also for antigen retrieval, the slides were autoclaved for 10 min in Tris-EDTA Buffer (pH = 9). After three times washing of the TMA slides in TBS, the slides were incubated for 20 min with 5 % sheep serum prepared in blocker protein (Dako, Denmark). Then, the slides were incubated overnight at 4 °C with the primary antibody (anti-dynamin 2 antibody, ab3457, Abcam, USA). Moreover, for isotype control, rabbit immunoglobulin (rabbit IgG) was used (both at a concentration of 100 ng/ml). After washing the slides, they were incubated for 1 h by ^TM^Mouse/Rabbit PolyVue HRP (DBS, USA) as the secondary antibody. Afterward, the slides were washed and then treated with 3,3’-diaminobenzidine (DAB) (Dako, Denmark) substrate as a chromogen for 20 min. After washing, the slides were counterstained with hematoxylin (Dako, Denmark). Finally, the slides were dehydrated, cleared in xylene, and then mounted. 

### Evaluation of immunostaining

In this study, the expression levels of dynamin 2 were evaluated using a semi-quantitative scoring system by two pathologists (M.R and Z.M) blinded to pathological information. A consensus was achieved for all the sample tissues. In addition, the intensity of staining was evaluated as 0, no staining; 1, weak; 2, moderate; and 3, strong staining. The percentage of positive cells was scored from 0 % to 100 % and then categorized according to the positive tumor cells as follows: < 25 % as 1, 25 %-50 % as 2, 51 %-75 % as 3, and > 75 % as 4. Finally, the histochemical score (H-score) was also obtained by multiplying the intensity score by the percentage of positive tumor cells, which yielded a range from 0 to 300. In this study, the H-scores were classified into three groups as follows: between 0 and 100 as group 1 (low expression), between 101 and 200 as group 2 (moderate expression), and between 201 and 300 as group 3 (high expression). In this regard, the mean of the three cores was calculated as the final score.

### Statistical analysis

SPSS software version 22.0 (Armonk, NY: IBM Co) was used for data analysis. Also, in this study, the categorical data were reported by N ( %) and quantitative data by mean (SD). Moreover, Pearson's χ2 test was used to analyze the significance of the association between the expression of dynamin 2 and clinicopathological parameters. A *p* value of < 0.05 was considered as statistically significant. 

## Results

### Patient's characteristics

Of 145 cases, 113 breast cancer patients were evaluated in this study, including 102 (90.3 %) IDC, 5 (4.4 %) ILC, 1 (0.9 %) combined IDC and ILC, 2 (1.8 %) metaplastic, and 3 (2.7 %) patients with other types of breast cancer. During this study, technical problems led to a loss of some cases. Notably, tumors in 50 (44.2 %) cases were on the right, in 60 (53.1 %) cases on the left, and in 3 (2.7 %) cases on the right and left (bilateral). In addition, the age variable followed an abnormal distribution; therefore, the median age of the patients was calculated as 49 years old (SD =13.6, ranged between 26 and 86). In this regard, 59 (52.2 %) patients were younger than 49 years old, and 54 subjects (47.8 %) were over 49 years old. Tumor sizes ranged from 1 to 17 cm, and 72 (63.7 %) cases were less than the mean size (≤ 4.5 cm) and tumor size was higher than the mean size (> 4.5 cm) in 41 (36.3 %) patients. In this study, 11 (9.7 %) tumors had a low histological grade (grade I), 52 (46.0 %) tumors were known as grade II, and 50 (44.2 %) tumors had a high histological grade (grade III). Moreover, LNI and VI were found in 78 (69.0 %) and 50 (44.2 %) cases, respectively. Accordingly, four (18.2 %) cases were stage I, 7 subjects (31.8 %) were stage II, and 11 patients (50.0 %) were stage III (Table 1(Tab. 1) and Supplementary Table 1).

### Expression of dynamin 2 in the patients with breast cancer and normal breast tissues

**Figure 1:**
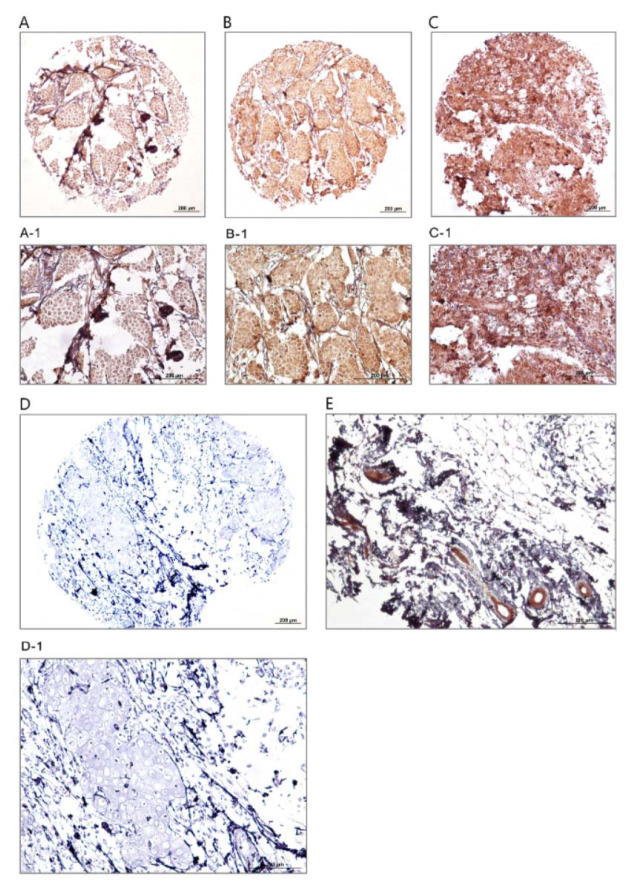
Immunohistochemical analysis of dynamin 2 expression in breast cancer samples and normal sample. Low expression (A, A-1), moderate expression (B, B-1), strong expression (C, C-1), negative (D, D-1), and normal tissue sample (E). Figures A, B, C, and D have magnification of 100 × and figures A-1, B-1, C-1, D-1, and E have magnification of 200 ×.

The expression levels of dynamin 2 molecule were assessed using IHC on TMA sections by three scoring methods as follows: intensity of staining, percentage of positive tumor cells, and H-score. The expression of dynamin 2 was observed at different intensities in the cell membrane, cytoplasm, and nucleus in the breast tumor tissue samples. Moreover, the normal tissues samples showed a lower expression level of dynamin 2 compared with cancerous tissues (Table 2(Tab. 2), Figure 1(Fig. 1)). Moreover, in this study, membranous expression of dynamin 2 was observed in 47 (41.6 %) cases, cytoplasmic expression in 108 (95.6 %) cases, and nuclear expression in 53 (46.9 %) cases. In addition, in normal tissues, membranous dynamin 2 expression was observed in 7 (70.0 %) cases, cytoplasmic expression in 9 (90.0 %) patients, and nuclear expression in 3 (30.0 %) tissues. Accordingly, the expression levels of this molecule are summarized in Table 2(Tab. 2).

### Association of nuclear dynamin 2 protein expression with the clinicopathological parameters

Pearson's χ2 test showed a statistically significant association between the increased expression level of nuclear dynamin 2 with the histological grade (H-score *P = 0.001*) and tumor stage in case of the staining intensity ***(****P = 0.05)*. In addition, a statistically significant association was observed between the higher expression levels of nuclear dynamin 2 and the patients' age (*H*-score *P = 0.03*).

Moreover, no association was found between the nuclear dynamin 2 expression and other tumor clinicopathological parameters. Also, the following parameters were obtained: tumor size (intensity P = 0.62; H-score P = 0.24), tumor types (intensity P = 0.59; H-score P = 0.80), tumor side (intensity P = 0.20; H-score P = 0.99), LNI (intensity P = 0.44; H-score P = 0.62), and VI (intensity P = 0.59; H-score P = 0.76) (Table 1(Tab. 1)).

### Association of cytoplasmic dynamin 2 protein expression with the clinico-pathological parameters

We found a significant difference in cytoplasmic dynamin 2 expression and tumor types in case of the staining intensity (*P = 0.003*). Moreover, a higher expression of cytoplasmic dynamin 2 expression was found in the IDC type.

Pearson's χ2 test exhibited no association among the cytoplasmic expressions of dynamin 2 and the histological grade (intensity P = 0.61; H-score P = 0.7), tumor stage (intensity P = 0.45; H-score P = 0.38), the patient's age (intensity P = 0.45; H-score P = 0.87), tumor side (intensity P = 0.14; H-score P = 0.42), tumor size (intensity P = 0.63; H-score P = 0.98), LNI (intensity P = 0.74; H-score P = 0.32), and VI (intensity P = 0.78; H-score P =0. 84) (Table 3(Tab. 3)).

### Association of membranous dynamin 2 protein expression with the clinicopathological parameters

At this stage, the results of Pearson's χ2 test revealed a statistically significant association between membranous dynamin 2 protein expression and VI in case of the staining intensity (*P = 0.02*).

Regarding this study, no significant association was found among the membranous dynamin 2 protein expression and the histological grade (intensity P = 0.5; H-score P = 0.62), tumor stage (intensity P= 0.86; H-score P = 0.72), age (intensity P = 0.23; H-score P = 0.42), tumor types (intensity P = 0.53; H-score P = 0.97), tumor side (intensity P = 0.43; H-score P = 0.63), tumor size (intensity P = 0.92; H-score P = 0.52), and LNI (intensity P = 0.88; H-score P = 0.38) (Table 4(Tab. 4)).

## Discussion

Despite the advances in breast cancer treatment, drug resistance and metastasis are known as major causes of mortality (Mansoori et al., 2019[27]). On the other hand, early diagnosis and the appropriate care according to the biological features of the disease are currently considered as the best approaches to treat the patients with breast cancer (Goldhirsch et al., 2003[10]). Therefore, finding new molecules for treatments tailored to the histological featurescan can lead to applying better therapeutic strategies and avoid excessive costs (Blumen et al., 2016[2]).

Dynamin 2 is a GTPase molecule consistently expressed in normal tissues (Ferguson and De Camilli, 2012[7]). Moreover, many studies have revealed that, dynamin 2 plays a role in some processes such as endocytosis (acting as a membrane fission molecule), morphogenesis, EMT, actomyosin contractions, and focal adhesion maturation, as well as those processes contributing to cancer progression (Chua et al., 2009[4]; Lamouille et al., 2014[23]; Edwards et al., 2016[5]; Jeong et al., 2006[15]; Gu et al., 2017[11]; Singh et al., 2017[40]). Furthermore, previous studies have shown over-expression of dynamin 2 in pancreatic, prostate, and cervical cancers (Eppinga et al., 2012[6]; Xu et al., 2014[42]; Lee et al., 2016[25]).

There are conflicting data on the role of dynamin expression in breast cancer (Piazza et al., 2009[31]; Rasmussen et al., 2010[35]; Khan et al., 2019[20]); therefore, it is necessary to investigate the dynamin 2 molecule in this cancer. 

In this study, the expression levels of dynamin 2 were investigated for the first time in a collection of 113 breast cancer tissue samples and normal cases as well as the clinicopathological parameters. In this regard, it should be noted that, the pattern of dynamin 2 expression in tumor cells was classified into nuclear, membranous, and cytoplasmic expressions and the analysis was also performed.

The present study, comparing the results of dynamin 2 expression in normal tissues with cancer tissues at three levels of nuclear, cytoplasmic, and membranous expression, showed that the cytoplasmic and nuclear dynamin 2 expressions have more increased in breast cancer tissues compared to normal tissues. Accordingly, these findings confirm the importance of the increased expression of this molecule in helping cancer to progress. Moreover, this study is in agreement with a study by Eppinga et al., which showed that 81 of 85 % of the patients had the increased dynamin 2 expression in pancreatic tumor tissues compared to normal tissues by analyzing the expression of dynamin 2 molecule in the human pancreatic tumors and histological features. In addition, histological analysis of metastatic pancreatic tumor outcomes showed that, dynamin 2 has elevated in 60 % of the metastatic tumors compared to benign tissues (Eppinga et al., 2012[6]). 

In our study, nuclear expression pattern of dynamin 2 indicated that the increased expression of dynamin 2 was associated with tumor stages. In addition, this study is in agreement with a study by Xu et al., who reported that dynamin 2 expression has significantly increased during the stages of prostate cancer (Xu et al., 2014[42]). In the explanation, dynamin has been shown to play an essential role in the endocytosis of membrane molecules and receptors such as ErbB2 and PDGFR (Giri et al., 2005[9]; Xia et al., 2011[41]; Sadowski et al., 2013[37]; Kranenburg et al., 1999[21]). In an experiment, the details of ErbB2 transferred from the cell surface to the nucleus in MCF7/ HER18 and MDA-MB-453, were investigated, and consequently, it was shown that transferring this molecule from the membrane surface to the cytoplasm is done by clathrin-mediated endocytosis. Nuclear translation of ErbB2 was also mediated by endosomal sorting. In fact, the intact vesicles derived from endocytosis of membranous ErbB2 enter the nucleus through the interaction between importin β1 bound to NLS (nuclear localization sequences) and Nup 358 (Nuclear pore). In addition, the results of electron microscopy and confocal immunofluorscence confirmed that, some molecules involved in this process such as dynamin 2 and early endosome antigen 1 (EEA1) colocalized with ErbB2 in the cytoplasm and nucleus. Furthermore, the use of dynamin 2 mutant abrogated nuclear translation of ErbB2 and showed the essential role of this molecule during this process (Giri et al., 2005[9]). Moreover, the presence of molecules involved in the process of endocytosis has been shown in the nucleus (Pyrzynska et al., 2009[33]). Therefore, dynamin 2 plays a role in cancer cells reprogramming through its role in endocytosis by transferring various molecules to the nucleus. Therefore, it can be concluded that translocation of dynamin 2 to the nucleus and the high level of dynamin 2 in the nucleus are indicators of the high level of molecules that can be transferred in the cells by endocytosis. Regarding the relationship between the high level of dynamin 2 in the nucleus and the tumor stage, it should be stated that, high levels of ErbB2 in the nucleus has been shown to act as a transcriptional coactivator of Stat3 (signal transducer and activator of transcription 3) for promoting the cyclin D1 expression and c-myc oncogene (Ralhan et al., 2010[34]; Xia et al., 2011[41]; Liu et al., 2018[26]). Therefore, dynamin 2 can cause cancer progress and also can cause an increase in its stages by transferring and activating such oncogenes. 

In the present study, a significant association was observed between the nuclear expression of dynamin 2 and the histological grade. Accordingly, grading is defined as the differentiation state of tumor cells compared to normal cells, and along with increasing the grade (1 to 3) of tumor is less differentiated (Bloom and Richardson, 1957[1]). As mentioned earlier, one of the results of nuclear ErbB2, which is transferred to the nucleus by endocytosis with the help of dynamin 2, is the activation of the oncogene molecules like c-myc (Liu et al., 2018[26]; Xia et al., 2011[41]). Accordingly, it has been shown that, overexpression of c-myc induces EMT in mammary epithelial cells (Cho et al., 2010[3]; Yin et al., 2017[45]). During the EMT process, the polarized epithelial cells undergo some multiple biochemical changes, loss of polarity, and acquiring the features of mesenchymal cells. In this manner, cancer cells lose their original properties and also acquire new features through the EMT, which is a process that is activated in high grade cancers and may play a role in the high grade cancers' generation (Kalluri and Weinberg, 2010[19]).

The analysis also revealed that, the increased membranous expression of dynamin 2 is associated with vascular invasion. In this regard, Lee et al. by examining the expression of dynamin 2 in the samples of 208 patients with early cervical cancer showed that, the expression of dynamin 2 is also related to tumor invasion (Lee et al., 2016[25]). Our results have shown that, VI can be considered as an indicator of aggressiveness for the patients with breast cancer. Because the phenomenon in which cells invade into the blood vessels is the initial and necessary step for cell metastasis, thus VI can predict metastasis. Regarding the relationship between the high levels of dynamin 2 membranous expression and VI, it should be stated that, an important feature of cancer cells is their high motility, which is a feature that requires the metastasis of cancer cells to other tissues. In addition, as it was mentioned earlier, one of the bestknown roles for dynamin 2 is its essential involvement in the formation of vesicles coated with clathrin during endocytosis (Oh et al., 1998[29]). Accordingly, the importance of this role of dynamin in helping the move of cancer cells and thus promoting the breast cancer progression and metastasis can be explained by the endocytosis of cadherin molecules. In this regard, it is a molecule that interconnects between epithelial cells, which is a process that helps the separation of cells from adjacent cells in the tissue (Paterson et al., 2003[30]). In agreement with this subject, it should also be stated that, dynamin 2 plays a role in actomyosin contractions involved in various functions including cell motility (Chua et al., 2009[4]; Jeong et al., 2006[15]). In addition, dynamin 2 molecule also helps the migration of cells through its role in the trafficking of Rac molecule, which is a small GTPase molecule of cytoskeletal actin regulating proteins that regulate the formation of lamellopodia (Matsubara and Bissell, 2016[28]; Schlunck et al., 2004[38]). Therefore, this molecule can promote metastases, and as its other role, dynamin 2 is the cross-linking of actin filaments, which characterizes the role of this molecule in focal adhesion formation (Gu et al., 2010[12]). Accordingly, focal adhesions are cell-matrix communication structures that are formed following the actin polymerization and the production of actomyosin contractile force, and are also associated with cancer progression. Moreover, the formation of this structure leads to the initiation of signaling pathways in cells associated with the survival, proliferation, and invasion of cancer cells (Matsubara and Bissell, 2016[28]; Geiger et al., 2009[8]). A study showed that, the treatment of actin filaments with dynamin 2 and cortactin results in the formation of thicker and longer bundles of actin and also increases the stability of actin bundles. In this way, it plays an important role in the development of cancer by helping the cancer cell migration (Yamada et al., 2016[44]). In confirmation of the role of dynamin 2 in cancer cells' movement, various studies have shown that, increasing the expression of dynamin 2 molecule increases the motility as well as metastatic capacity of cancer cells, and inhibition of this molecule results in a decreased metastatic capacity of cancer cells (Razidlo et al., 2013[36]). 

The limitation of our study was the unavailability of the patients' survival information, as having this data would help in expanding the findings and also determining the prognosis of the disease.

## Conclusions

Overall, our results showed that, the increased expression of dynamin 2 in breast cancer tissue samples rather than normal cases and nuclear expression level of dynamin 2 molecule is associated with the tumor stages and histological grades of tumor cells. In addition, the level of dynamin 2 membranous expression is associated with the increased tumor invasion, which results in more advanced disease and may be considered as an indicator of the extent of invasion of breast cancer cells into blood vessels, metastases, and progression. The obtained results also show that, the level of cytoplasmic expression of this molecule is associated with the disease type. Therefore, evaluation of the dynamin 2 expression patterns at three levels of nuclear, membranous, and cytoplasm is useful to predict the tumor invasiveness and progression of disease. Further studies are needed to identify the mechanism of action of this molecule as well as investigating this molecule as a therapeutic target in breast cancer.

## Notes

Zahra Madjd and Roya Ghods (Oncopathology Research Center, Iran University of Medical Sciences, Tehran, Iran; Department of Molecular Medicine, Faculty of Advanced Technologies in Medicine, Iran University of Medicine Sciences, Tehran, Iran; Tel/Fax: +982186704837, E-mail: ghods.ro@iums.ac.ir, rghods77@yahoo.com) contributed equally as corresponding authors.

## Acknowledgement

This work was supported by a grant from Iran University of Medical Sciences (No 96-02-126-30939). 

## Conflict of interest

All authors declare that there is no conflict of interest.

## Supplementary Material

Supplementary material

## Figures and Tables

**Table 1:**
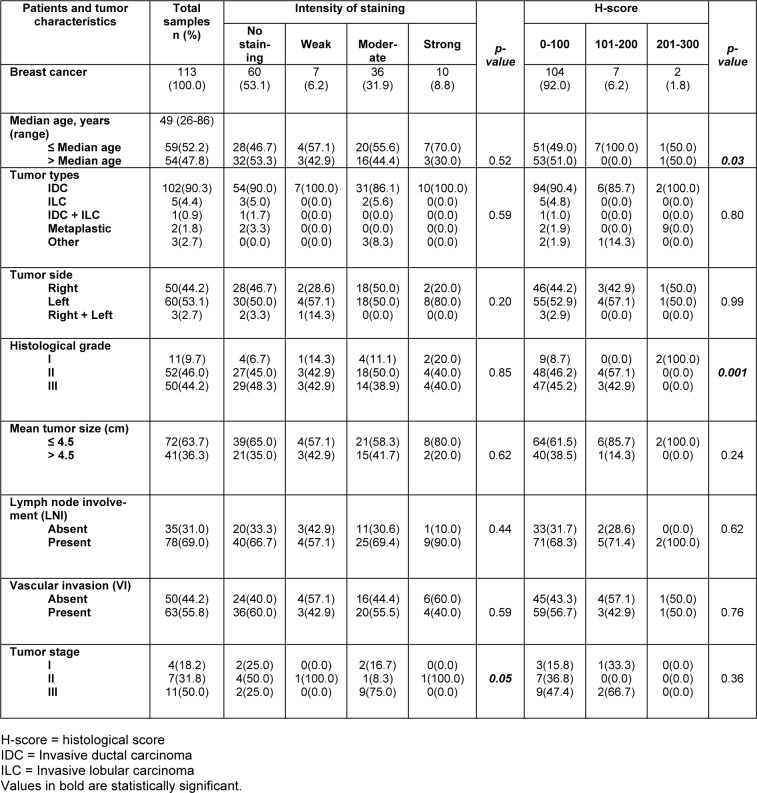
The association between nuclear dynamin 2 expression and clinicopathological parameters of breast cancer (intensity of staining and H-score; *p-value*; Pearson's χ2 test)

**Table 2:**
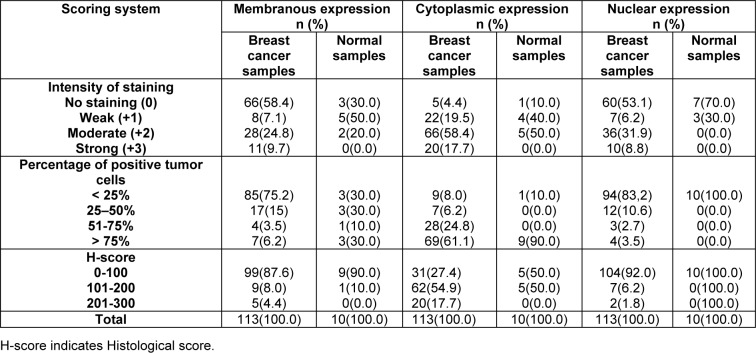
Dynamin 2 expression (intensity of staining, percentage of positive tumor cells, and H-score) in breast cancer and normal samples

**Table 3:**
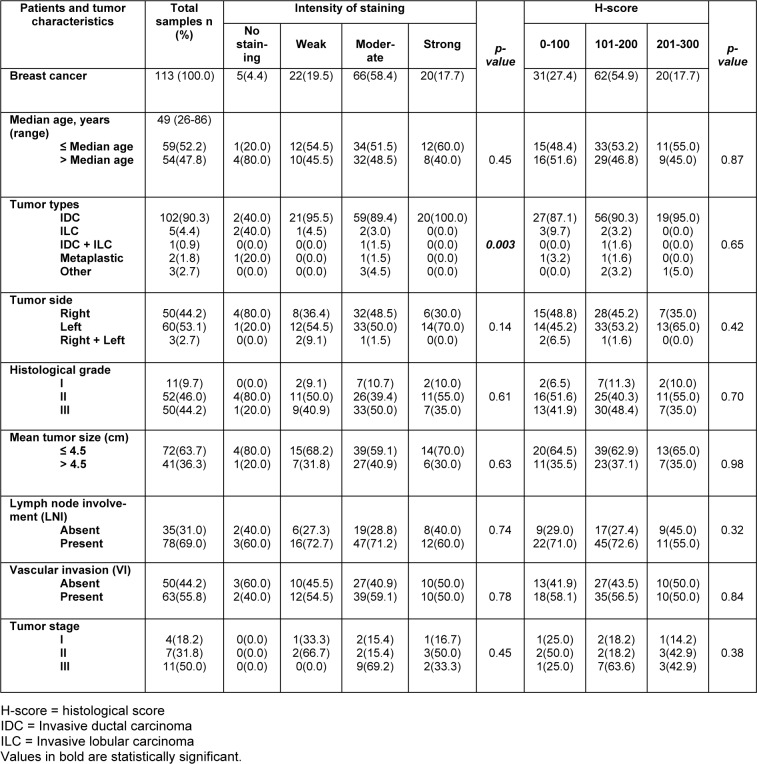
The association between cytoplasmic dynamin 2 expression and clinicopathological parameters of breast cancer (intensity of staining and H-score; *p-value*; Pearson's χ2 test)

**Table 4:**
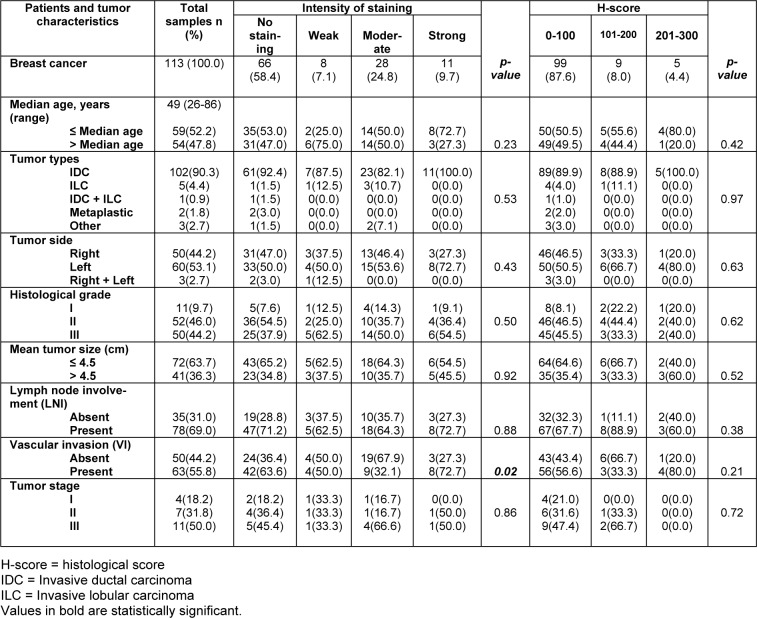
The association between membranousdynamin 2 expression and clinicopathological parameters of breast cancer (intensity of staining and H-score; *p-value*; Pearson's χ2 test)
