# Supplementary material for: Overexpression and translocation of dynamin 2 promotes tumor aggressiveness in breast carcinomas
Source: EXCLI J. 2020 Oct 29;19:1423–35. doi: 10.17179/excli2020-2762 (PMC7689243; doi:10.17179/excli2020-2762)
Supplement: Supplementary material [file EXCLI-19-1423-s-001.pdf]

EXCLI Journal 2020;19:1423-1435 – ISSN 1611-2156
Received: August 05, 2020, accepted: October 10, 2020, published: October 29, 2020

Supplementary material to:

OVEREXPRESSION AND TRANSLOCATION OF DYNAMIN 2 PROMOTES TUMOR AGGRESSIVENESS IN BREAST CARCINOMAS

Roya Sajed[1, 2], Leili Saeednejad Zanjani[2], Mandana Rahimi[3], Maryam Mansoori[1, 2], Amir-Hassan Zarnani[4, 5,] Zahra Madjd*[2, 1], Roya Ghods*[2, 1]

[1] Department of Molecular Medicine, Faculty of Advanced Technologies in Medicine, Iran University of Medicine Sciences (IUMS), Tehran, Iran
[2] Oncopathology Research Center, Iran University of Medical Sciences (IUMS), Tehran, Iran
[3] Hasheminejad Kidney Center, Pathology Department, Iran University of Medical Sciences (IUMS), Tehran, Iran
[4] Department of Immunology, School of Public Health, Tehran University of Medical Sciences (TUMS), Tehran, Iran
[5] Reproductive Immunology Research Center, Avicenna Research Institute (ACECR), Tehran, Iran

* **Corresponding authors:** Dr Roya Ghods, PhD, Professor Zahra Madjd, MD PhD, Oncopathology Research Center, Iran University of Medical Sciences, Tehran, Iran; Department of Molecular Medicine, Faculty of Advanced Technologies in Medicine, Iran University of Medicine Sciences, Tehran, Iran;
Tel/Fax: +982186704837; E-mail: ghods.ro@iums.ac.ir; rghods77@yahoo.com;
Tel/Fax: +982188622608; E-mail: majdjabari.z@iums.ac.ir; zahra.madjd@yahoo.com

http://dx.doi.org/10.17179/excli2020-2762

This is an Open Access article distributed under the terms of the Creative Commons Attribution License (http://creativecommons.org/licenses/by/4.0/).

EXCLI Journal 2020;19:1423-1435 – ISSN 1611-2156
Received: August 05, 2020, accepted: October 10, 2020, published: October 29, 2020

Supplementary Table 1: Patients and pathological characteristics of breast cancer (BC) tumors

Patients and tumor characteristics	Total samples N (%)
**Median age, years (range)**	49 (26-86)
**≤ Median age**	59 (52.2)
**> Median age**	54 (47.8)
**Tumor types**	
**IDC**	102 (90.3)
**ILC**	5 (4.4)
**IDC + ILC**	1 (0.9)
**Metaplastic**	2 (1.8)
**Others**	3 (2.7)
**Tumor side**	
**Right**	50 (44.2)
**Left**	60 (53.1)
**Right + Left**	3 (2.7)
**Histological grade**	
**I**	11 (9.7)
**II**	52 (46.0)
**III**	50 (44.2)
**Mean tumor size (cm)**	4.5
**≤ Mean**	72 (63.7)
**> Mean**	41 (36.3)
**Lymph node involvement (LNI)**	
**Absent**	35 (31.0)
**Present**	78 (69.0)
**Vascular invasion (VI)**	
**Absent**	50 (44.2)
**Present**	63 (55.8)
**Tumor stage**	
**I**	4 (18.2)
**II**	7 (31.8)
**III**	11 (50.0)
**Total**	113 (100.0)

IDC = Invasive ductal carcinoma
ILC = Invasive lobular carcinoma
